# Libya, HIV, and open communication

**DOI:** 10.1186/1742-4690-3-99

**Published:** 2006-12-28

**Authors:** Kuan-Teh Jeang

**Affiliations:** 1 The National Institutes of Health, Bethesda, MD, USA

## Abstract

This year-end editorial discusses several points including the recent Libyan verdict sentencing five Bulgarian nurses and a Palestinian doctor to death for allegedly infecting 426 children with HIV. It also comments on the role played by open communication for bridging cultural misunderstandings and summarizes briefly *Retrovirology's *progress in 2006.

## Libya, once again

More than two years ago in September 2004, I wrote an editorial entitled "Mohmmar Qadaffi, open access, and retrovirology" [1]. At that time, I did not imagine that the topic of Libya would ever emerge in another of my editorials. Yet, today I again comment on Libya, a country in which I grew up from the ages of five to twelve, driven by a recent verdict sentencing five Bulgarian nurses and a Palestinian doctor to death for allegedly infecting 426 children with HIV.

The Libyan-HIV case began in 1998 when Bulgarian nurses arrived to work in a Benghazi children's hospital. Shortly, thereafter, many children in the hospital became seropositive for HIV. What caused these happenings is being contested. The Libyan government and distressed family members say that the Bulgarian medical workers and the Palestinian doctor are causal. Others argue that the cause is less nefarious and is likely explained by poor hygiene and sterilization practices at the Libyan hospital. There are recent scientific data based on virus genetics which indicate that the HIV infection in the Libyan children started prior to March 1998, when the accused and now convicted medical staff arrived [2]. Such findings would exonerate the accused, and cry out for the correction of a miscarriage of justice.

What next? First, as with all man-made impasses, how should one move to a successful resolution? Second, how does one learn from this lesson to prevent future similar recurrences? On the first, 114 Nobel laureates have weighed in providing a consensus advice [3]. This advice should be followed. Ultimately, substantive resolution might have to come from intervention by Qadaffi to commute the sentences. Alternatively, there could be a large monetary out-of-court settlement that assuages the 400+ aggrieved families.

On the question as to what we can learn, a couple of comments could be made. The difficulty in settling the Libyan-HIV problem seemingly mirrors other quagmires such as the US-Iraq, the US-Iran, and the US-North Korea disputes. All these situations involve opposing parties who see things in diametrically opposite ways. Perhaps, one way whereby a gulf of misunderstanding and mistrust can be bridged is if we spend more time prior to conflicts communicating with and engaging each other. This seems to be common sense, but the surprising matter is that we do not routinely follow our "common sense". For example, the US has trade disputes fairly frequently with Europe and Japan, and yet each fracas seems to be worked out over time. Perhaps it is because in those settings, the disparate parties can speak a "common language", and thus constructive dialogues can be promulgated. Contrast this with a recent finding from the Iraq Study Group (ISG). One minor, but telling point, raised by the ISG was that out of the 1000 persons currently working at the US Embassy in Baghdad, only 6 speak Arabic. Yes, only 6! How then does one conduct diplomacy without speaking a language that can be understood by one's clients or opponents? In a similar vein, the facts of the case aside, would it not be easier to persuade the Libyans if we spoke "Libyan" (i.e. if some of us doing the persuading had historical, established or ongoing relationships with Libyan colleagues)? Sadly, I may be one of only a very few HIV-1 researchers who have ever been to Libya, let alone have lived there and am acquainted with the culture. Hindsight is indeed 20–20 in vision. Nonethelesss, going forward, if we choose to nurture selective relationships while dismissing other equally worthwhile opportunities, then we should not necessarily be surprised if some parties refuse to listen to us and view us with mistrust.

## Open dialogue and *Retrovirology*, a personal take

I was born in Taiwan, but left there after 5+ years to live in Libya, and then later I came to the US at the age of 12. Many Americans look at me and instinctively see me as Chinese. Chinese do not view me as being sufficiently Chinese but as American in Asian clothing. And of course, my Libyan schoolmates would not recognize me as one of them. However, rather than being distressed about a mixed "national identity", I am comfortable with having lived in many lands, having met many people, and having spoken in several cultures. There are many reasons why I wanted to start *Retrovirology*, but the open access, the freely accessible "open dialogue" format, was a key attraction for me.

*Retrovirology *is a small example of how information and ideas should be distributed in the 21^st ^century. In science there are also "Libyan communities" which are out-of-the loop and do not have equal access as others to subscription-based literature. But every "Libyan" scientist is on equal footing with a "London" or "Bethesda" colleague when it comes to accessing *Retrovirolog*. If "Libya", "London" and "Bethesda" have the same access and read the same materials, then is it not a better bet that they might understand each other more than if each had different access and read differently.

Two other items that *Retrovirology *brings to "Libyan" communities are meeting proceedings and meeting reviews [4-7]. Many of us take for granted attendance at meetings as a routine part of science; however, many others do not have the resources or in some cases the permission to go to such gatherings. As much as we can and are asked to do, *Retrovirology *is interested in publishing the full abstracts of all talks and posters presented at international meeting such as that we did recently for the 2006 Institute of Human Virology meeting [8].

## End-of-year thanks

In 2005, *Retrovirology *published more papers than in we did in 2004. In 2006, we again published more than in 2005. *Retrovirology *papers are being cited with frequency; two of our more visible examples, Omoto *et al*. [9] and Sebastian and Luban [10], have been cited to date 27 and 24 times respectively. For our progress, *Retrovirology *thanks our authors, our reviewers, our editorial board, and our Associate editors [11].
